# Generation of heritable germline mutations in the jewel wasp *Nasonia vitripennis* using CRISPR/Cas9

**DOI:** 10.1038/s41598-017-00990-3

**Published:** 2017-04-19

**Authors:** Ming Li, Lauren Yun Cook Au, Deema Douglah, Abigail Chong, Bradley J. White, Patrick M. Ferree, Omar S. Akbari

**Affiliations:** 1 Department of Entomology and Riverside Center for Disease Vector Research, Institute for Integrative Genome Biology, University of California, Riverside, Riverside, CA 92521 USA; 2 W.M. Keck Science Department, Claremont McKenna, Pitzer and Scripps Colleges, 925 Mills Avenue, Claremont, CA 91711 USA

## Abstract

The revolutionary RNA-guided endonuclease CRISPR/Cas9 system has proven to be a powerful tool for gene editing in a plethora of organisms. Here, utilizing this system we developed an efficient protocol for the generation of heritable germline mutations in the parasitoid jewel wasp, *Nasonia vitripennis*, a rising insect model organism for the study of evolution, development of axis pattern formation, venom production, haplo-diploid sex determination, and host–symbiont interactions. To establish CRISPR-directed gene editing in *N*. *vitripennis*, we targeted a conserved eye pigmentation gene *cinnabar*, generating several independent heritable germline mutations in this gene. Briefly, to generate these mutants, we developed a protocol to efficiently collect *N*. *vitripennis* eggs from a parasitized flesh fly pupa, *Sarcophaga bullata*, inject these eggs with Cas9/guide RNA mixtures, and transfer injected eggs back into the host to continue development. We also describe a flow for screening mutants and establishing stable mutant strains through genetic crosses. Overall, our results demonstrate that the CRISPR/Cas9 system is a powerful tool for genome manipulation in *N*. *vitripennis*, with strong potential for expansion to target critical genes, thus allowing for the investigation of several important biological phenomena in this organism.

## Introduction

Hymenopteran insects, including all ants, bees, and wasps, represent one of the most prominent insect orders, occupying roughly 8% of all described species on earth^1^. The parasitoid wasp *Nasonia vitripennis* is one of the most tractable and comprehensively studied hymenopterans genetically^2^, owing to its overall ease of laboratory use, its short generation time (roughly ~2 weeks), tolerance for inbreeding, and straightforward rearing. Like all other hymenopterans, *N*. *vitripennis* utilizes a haplodiploid sex determination system by which haploid males develop parthenogenetically from unfertilized eggs while diploid females develop from fertilized eggs^2^. Interestingly, this mode of sex determination makes *N*. *vitripennis* and other members of the clade vulnerable to manipulation by microbial and genetic parasites. For example, *Arsenophonus nasoniae*, a natural bacterial endosymbiont of *N*. *vitripennis*, effectively kills male progeny by manipulating key components of the mitotic machinery required specifically for early male embryonic development^3^. This male-killing results in significantly biased sex ratios favoring females, thereby benefiting the bacteria as they are transmitted solely from infected mother to offspring^4^. In addition to sex ratio-distorting bacteria, other genetic agents can influence the sex ratios of hymenopteran insects. For example, although the genome of *N*. *vitripennis* naturally harbors five chromosomes, some individuals have been discovered to also contain a sixth, supernumerary (B) chromosome termed paternal sex ratio (PSR)^5^. PSR is paternally transmitted through the sperm and acts by eliminating the haploid genome, thereby converting what should be diploid females into haploid PSR transmitting males, thereby making it a remarkable and potent selfish chromosome^5, 6^. While progress has been made toward uncovering PSR-expressed transcripts^7^, the mechanism of action of this B chromosome in the *N*. *vitripennis* genome largely remains to be elucidated.

The last decade has experienced a rapid increase in the genetic toolkit to study the biology of *N*. *vitripennis* and its interesting interactions with bacterial symbionts and genetic parasites. For example, the availability of its high-resolution sequenced genome^8, 9^, and several recent tissue-specific gene expression studies, together have provided a wealth of developmental gene expression information to be functionally analyzed^7, 10, 11^. Furthermore, methods to functionally disrupt gene expression relying on RNA interference (RNAi) by injecting *in vitro* transcribed dsRNA into either female pupae^12^ or larvae^13^ have advanced capabilities of performing reverse genetics on this organism. Altogether, these features have rendered *N*. *vitripennis* as a burgeoning model organism^13–16^ for studying complex genetic, cellular and developmental processes including venom production^17, 18^, sex determination^19^, host symbiont interactions^3, 20^, evolution and development of axis pattern formation^21–24^, and development of haplodiploidy^24^.

While *N*. *vitripennis* has many amenable experimental tools and resources described above, to date there have been no successful methods developed that allow for direct gene mutagenesis in this organism. This absence can, in part, be attributed to the difficulty in using previous gene disruption technologies, e.g. TALENs and ZNFs^25^, in addition to a lack of detailed published protocols for easily performing embryonic microinjection in *N*. *vitripennis*. To overcome these significant limitations, here we have employed the CRISPR-Cas9 (clustered regularly interspaced short palindromic repeats) gene editing system in *N*. *vitripennis*. As a part of this system we developed an effective method for pre-blastoderm stage embryonic microinjection in this organism. We report robust embryonic survival rates following embryo microinjection, and high mutagenesis rates of the conserved eye marker gene *cinnabar* in surviving CRISPR-Cas9 injected individuals. Overall, we demonstrate an efficient, effective, inexpensive, and straightforward CRISPR-Cas9 heritable gene disruption approach for *N*. *vitripennis*, and to our knowledge this study represents one of the first gene disruption-based techniques conducted in a hymenopteran insect.

## Results

### Development of an CRISPR/Cas9 embryo microinjection protocol

**Figure 1:**
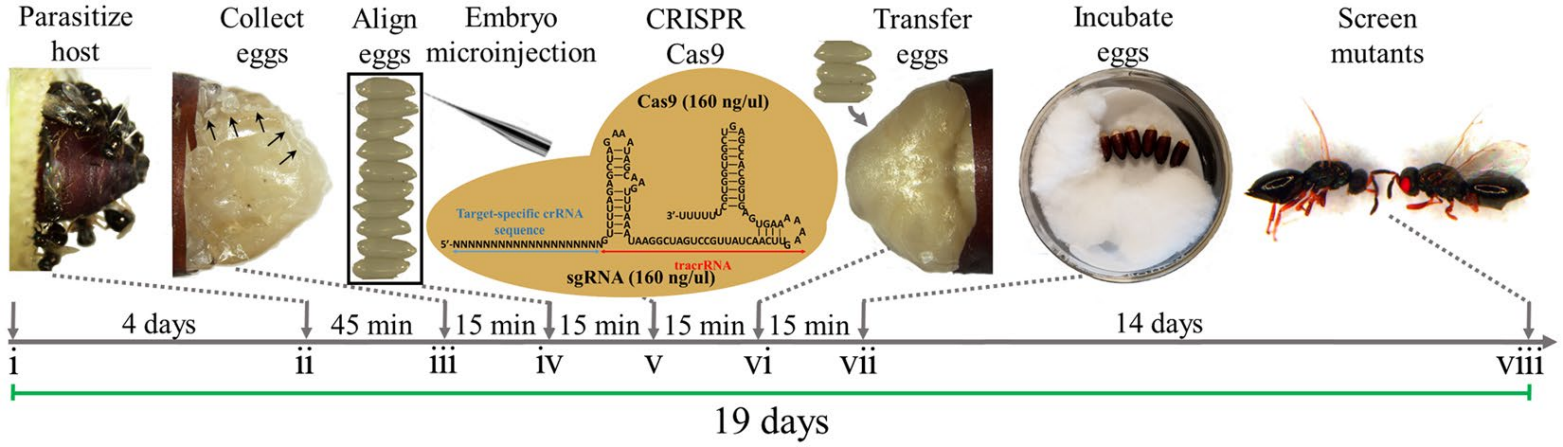
Schematic of *Nasonia vitripennis* embryo collection and CRISPR/Cas9 microinjections. Adult *Nasonia vitripennis* were mated for 4 days (i), then were supplied with a flesh fly host pupa, *Sarcophaga bullata*, for female parasitization for 45 minutes (ii). Embryos were then collected from the host (iii), aligned (iv), and injected with CRISPR/Cas9 components (v). Injected embryos were then gently placed back into the host (vi) for development (14 days) (vii), and when the adults emerged from the host they were subsequently screened for CRISPR/Cas9 induced mutations in target gene (viii). This entire procedure takes roughly 19 days to complete.

For delivery of CRISPR-based reagents we initially established efficient techniques for egg collection, pre-blastoderm stage embryo microinjection, and subsequent rearing and genetics, before proceeding. Development of these pre-blastoderm stage embryo microinjection techniques, combined with subsequent rearing and genetics, was essential to access the germline and enable heritable genome editing in an effort to establish stable mutant strains. Briefly, as illustrated in Fig. 1, our techniques involved (i) permitting male and female adults to mate (~4 days), (ii) supplying fresh host fly pupae (*Sarcophaga bullata*) to mated females for oviposition (~45 minutes), (iii) carefully opening the parasitized host pupae to collect pre-blastoderm stage wasp embryos (~15 minutes), (iv) aligning these embryos on sticky tape (~15 minutes), (v) micro-injecting embryos with CRISPR/Cas9 components (~15 minutes), (vi) carefully placing injected embryos back into the pre-stung hosts for proper development (~15 minutes), (vii) and transferring the parasitized hosts harboring the CRISPR/Cas9 injected embryos into a humidified chamber with roughly 70% relative humidity to prevent dehydration of the embryos/host (~15 minutes). These parasitized hosts were then incubated for roughly 14 days to permit the *N*. *vitripennis* embryos to complete development, and once the injected adults emerged from the host (viii), we isolated, mated and screened these individually for the presence of mutations (see Methods and Supplemental Methods for a comprehensive, step-by-step protocol). Remarkably, this entire protocol, from mating, to injecting, to hatching of injected individuals takes roughly 19 days for completion.

To initially test this injection protocol, we measured and compared the survival rates (to adulthood) of non-injected wasp embryos (*i*.*e*., embryos removed from host, lined up on slide, then carefully placed back into host) to embryos injected with only purified water (*i*.*e*., embryos removed from host, lined up on slide, injected with water, then carefully placed back into hosts). We found our survival rates to be quite robust for both non-injected embryos (92%), and for embryos injected with only water (76%).

### Identification of CRISPR/Cas9 target sites

**Figure 2:**
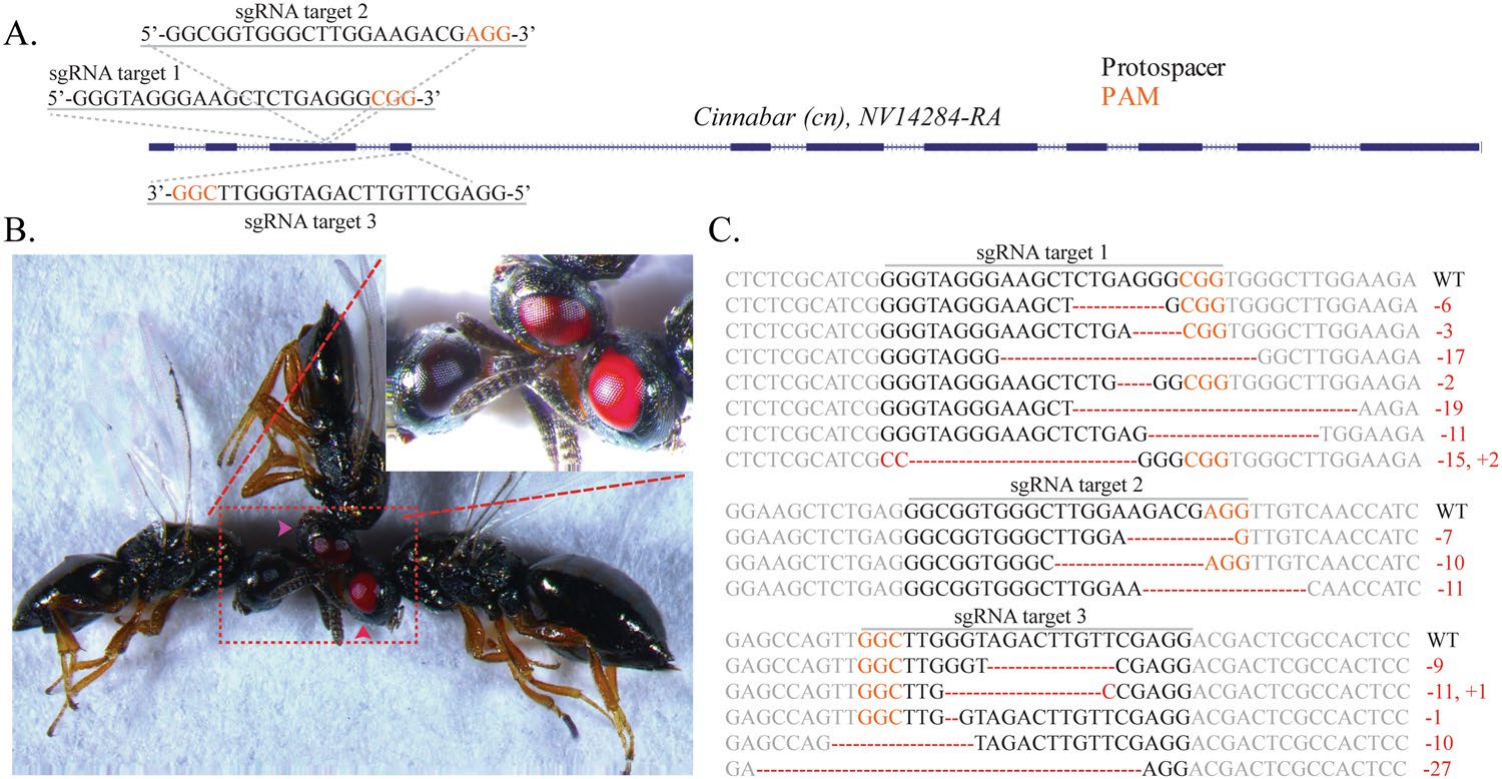
CRISPR/Cas9 target sites, mutant *cinnabar* phenotypes, and sequence disruption confirmations. Three independent sgRNAs were designed to target *cinnabar* in either exon 3 (sgRNA target 1 & 2) or exon 4 (sgRNA target 3) as depicted (**A**). Following embryo microinjection, surviving *cinnabar* mutant G0 adult wasps were readily observable with a light microscope by simply observing eye color phenotypes. Black eyes are wild-types, while bright red (younger - within a few days of emergence; indicated by red arrowhead) and red (older - roughly a week postemergence; indicated by purple arrowhead) are mutants with different age (**B**). Many mutants for each sgRNA were established and deletions and insertions were readily detected via sequencing. The black cn+ pigmentation appears normally during pupal development. However, in CRISPR-induced *cn* mutants, the garnet-colored eye never undertakes a black phenotype and can be easily seen in adults. These mutant phenotypes can also be scored in late pupal stages (not shown) (**C**). PAM sequences (NGG) are indicated in orange, and *cn* gene disruptions resulting from insertions/deletions are indicated in red.

To establish an efficient CRIPSR/Cas9 based genome editing platform for *N*. *vitripennis* we targeted the conserved dominant *cinnabar* (*cn*) gene (NV14284), which encodes for kynurenine hydroxylase, an enzyme involved in ommochrome biosynthesis^26^. Importantly, mutations in this gene result in distinct, scorable eye-color phenotypes when mutated in many organisms^27, 28^, including *N*. *vitripennis* when silenced via larval RNAi^13^, thereby making it an optimal choice for the development and testing of a CRIPSR/Cas9 based gene mutagenesis technique in this organism. To disrupt this gene using CRISPR/Cas9, we designed several short guide RNAs (sgRNAs) to target either the third (sgRNA target sites 1 & 2) or the fourth (sgRNA target site 3) exons of the *cn* gene (Fig. 2A). To define these specific exonic sgRNA genomic target sites we considered several factors. Firstly, we utilized available *N*. *vitripennis* transcriptional databases (www.vector.caltech.edu) to confirm *cn* RNA expression of the putative target regions^7, 10^. Secondly, we searched both sense and antisense strands of the *cn* exon sequences of interest for the presence of the NGG protospacer-adjacent motifs (PAMs) utilizing CHOPCHOP v2 software^29^ and local sgRNA Cas9 package^30^. Thirdly, to minimize potential off-target effects, we confirmed specificity of our sgRNAs using publicly available bioinformatic tools^31^ and selected the most specific sgRNAs within our specified target region.

### Mutagenesis of the cinnabar gene is sgRNA/Cas9 dose dependent

To determine the optimal sgRNA/Cas9 concentrations for efficient disruption of *cn*, sgRNA-1 was chosen as a standard. We combined a variety of concentrations of sgRNA-1 (0, 20, 40, 80, 160, and 320 ng/ul) with the Cas9 protein (0, 20, 40, 80, 160, and 320 ng/ul) and found that the survival rate of the injected embryos, and the efficiency of mutagenesis mediated by CRISPR/Cas9, were dose-dependent (Table 1). These components also had an inverse relationship to each other; as the increased concentration of sgRNA and Cas9 protein lead to the increased proportion of red eye mutant adults (up to 60% of adult G0 survivors), the survival rate of injected eggs concomitantly decreased (Table 1). Therefore, we used the optimal combination of 160 ng/ul sgRNA and 160 ng/ul Cas9 protein as the working concentration for subsequent experiments.

**Table 1:** Effect of sgRNA and Cas9 protein concentration on *N*. *vitripennis* survival and mutagenesis.

sgRNA-1	Cas9	Total embryos	Adult Survivors			Mosaic (%)		
			♂	♀	Total (%)	M (%)	F (%)	Both (%)
No injection	No injection	100	66	26	92 (92)	0 (0)	0 (0)	0 (0)
Water	Water	100	44	32	76 (76)	0 (0)	0 (0)	0 (0)
20 (ng/ul)	20 (ng/ul)	100	34	34	68 (68)	0 (0)	0 (0)	0 (0)
40	40	100	30	32	62 (62)	4 (13)	0 (0)	4 (6)
80	80	100	24	22	46 (46)	6 (25)	0 (0)	6 (13)
160	160	100	16	22	38 (38)	5 (31)	3 (14)	8 (21)
320	320	100	10	10	20 (20)	6 (60)	6 (60)	12 (60)

To expand these studies and test other CRISPR/Cas9 target sites of *cn*, we injected an increased number of embryos (N = 300) for each sgRNA/Cas9 combination, using our optimized sgRNA/Cas9 concentrations 160 ng/ul (Table 1), and had survival rates ranging from 22–27% of total embryos injected. From these injections, we discovered that 32% and 36% of injected survivor G0 *N*. *vitripennis* adults displayed the *cn* mutant phenotypes (*i*.*e*., complete bilateral red eyes, Fig. 2B) following microinjection with either sgRNA-1/Cas9, or sgRNA-3/Cas9 complexes, respectively (Table 2). However, lower mutagenesis efficiency (10%) was observed when sgRNA-2 was utilized, presumably resulting from inefficiency of sgRNA-2 (Table 2). Furthermore, in some instances we observed surviving G0 adults expressing a variegated (*i*.*e*., mottled) red/black eye phenotype (not shown), or in some cases, unilateral disruption (*i*.*e*., one complete black eye and one complete red eye in the same individual, not shown), as opposed to complete bilateral red eyes (mutant, Fig. 2B) or complete bilateral black eyes (WT, Fig. 2B), which we attributed to gene editing occurring in some nuclei at nuclear divisions past the first embryonic mitotic division (e.g. 2-nucleus embryo stage or later). Overall, these results strongly demonstrate the efficiency of the CRISPR/Cas9 system in *N*. *vitripennis* targeting multiple independent sites.

**Table 2:** Summary of the injection and mutagenesis mediated by independent sgRNAs in *N*. *vitripennis*.

sgRNA	#Injected	Adult Survivors			Mosaic (%)		
		♀	♀	Total (%)	M (%)	F (%)	Both (%)
sgRNA-1	300	50	24	74 (25)	16 (32)	8 (33)	24 (32)
sgRNA-2	300	46	39	82 (27)	4 (8)	4 (11)	8 (10)
sgRNA-3	300	30	36	66 (22)	10 (33)	14 (39)	24 (36)

### Transmission of mutations to subsequent generations

Germline transmission of the CRISPR/Cas9 mutations to subsequent generations is essential for establishing stable mutant stocks. Given that all hymenopteran insects have haplodiploid sex determination with no heteromorphic sex chromosomes, and the widespread mode of reproduction is arrhenotoky, by which males develop from unfertilized eggs and are haploid while females develop from fertilized eggs and are diploid, these factors had to be taken into consideration when designing the genetic crossing schemes to homozygose mutant strains. Therefore, to test the germline transmission efficiency of the mutations generated by CRISPR/Cas9, and to establish homozygous mutant stocks, four crossing strategies were employed from 300 G0 individuals injected with sgRNA-1-3 (Table 3). Overall, the results indicated that mutations are produced within the germline and transmitted to the subsequent generations with very high efficiency (e.g. 100% all male G1 offspring contained the mutant eye with crossing strategy D) and stable 100% mutant (male and female) producing lines could be produced by the G3 generation with the various crossing strategies. Together, these results indicated that the mutations had been efficiently transmitted into the germline and can be maintained in subsequent generations. Additionally, induced mutations can be obtained from either the G0 male or female parental direction.

**Table 3:** Summary of G1, G2 and G3 phenotypes of *N*. *vitripennis* with different crossing strategies.

Crossing strategy	G1 adult phenotype		G2 adult phenotype		G3 adult phenotype	
	♂^W−^ (%)	♀^W−^ (%)	♂^W−^ (%)	♀^W−^ (%)	♂^W−^ (%)	♀^W−^ (%)
A	133 (92)	31 (95)	773 (100)	336 (100)	3741 (100)	1159 (100)
B	0 (0)	0 (0)	71 (31)	62 (17)	659 (100)	271 (100)
C	12 (7)	0 (0)	93 (35)	57 (22)	1318 (100)	577 (100)
D	52 (100)	0 (0)	0 (0)	0 (0)	1173 (24)	227 (33)

For cross strategy A, G0 mutant ♂ X G0 mutant ♀, G1 mutant ♂ X G1 mutant ♀, G2 mutant ♂ X G2 mutant ♀. For cross strategy B, G0 mutant ♂ X wild type ♀, G1 self-cross, G2 mutant ♂ X G2 mutant ♀. For cross strategy C, G0 mutant ♀ X wild type ♂, G1 self-cross, G2 mutant ♂ X G2 mutant ♀. For cross strategy D, G0 mutant ♀ unmated, G1 mutant ♂ X wild type ♀, G2 mutant ♂ X G2 mutant ♀.

Finally, to conclusively confirm the phenotypic defects described above were due to the genomic mutagenesis of the *cn*, genomic DNA was extracted from several independent mutant G2 lines and used as the template to PCR amplify the genomic DNA fragment containing the *cn* sgRNA target sites. The sequencing results confirmed the insertions/deletions in *cn* for all three sgRNA target sites tested (Fig. 2C). Additionally, all sequenced lesions disrupted by the sgRNA target sequences generated deletions ranging from the loss of a single nucleotide to the loss of 27 nucleotides, and in some cases adding additional nucleotides around the targeted sites, in all cases disrupting gene function (Fig. 2C).

## Discussion

Over the past decade or so, several important genetic, genomic, and cell biological studies have been conducted in the jewel wasp *N*. *vitripennis* ^19, 32–35^. These studies have been facilitated by the development of several important experimental resources including a high resolution genome sequence^8^, several genome wide transcriptional profiling studies^7, 10^, procedures for performing embryonic *in situ* hybridizations to detect spatial patterns of mRNA expression^34^, and systemic, parental RNAi which can be used in certain tissue contexts to study gene function using reverse genetics^12, 13^. Together these tools and others have progressively contributed to *N*. *vitripennis* becoming a preferred experimental system for hymenopteran-related biology. Notwithstanding these effective tools and resources, what has been lacking in *N*. *vitripennis* is a means for performing directed, heritable gene mutagenesis, which would facilitate efficient *in vivo* functional analysis of candidate genes in this species. To address this limitation, we tested whether the CRISPR/Cas9 system could be exploited as an effective gene editing platform in *N*. *vitripennis*. Overall our results demonstrate that the CRISPR/Cas9 system works efficiently in this organism; as a proof of principle we used this system to disrupt a conserved eye-pigmentation gene *cn*, utilizing several different sgRNAs with mutation rates up to 60%. Additionally, we found that these mutations were heritable, allowing us to homozygous them and establish stable mutant stocks.

Our study contains a few important caveats worth consideration. For example, we noticed that the efficiency of mutagenesis mediated by CRISPR/Cas9 and the survival rate of *N*. *vitripennis* injected embryos were sgRNA- and Cas9 protein-concentration dependent. Injected eggs with high concentrations of sgRNA and Cas9 combinations had higher mutagenesis rates but lower survival rates. A similar effect was also reported in other insects^36, 37^, indicating that, on one hand, the concentration of injected sgRNA and Cas9 protein should be high enough to generate bi-allelic mutations to establish stable mutant populations; however, on the other hand, high concentration of sgRNA and Cas9 protein may cause toxic effects to the insects, thereby making it difficult to recover surviving mutant individuals. Our experiments suggest that an intermediate concentration of 160 ng/ul for both the Cas9/sgRNA components achieves a moderate mutation rate while minimizing reduction of survivorship. We also noticed that the efficiency of cleavage is target site-dependent because each sgRNA we tested had a different cleavage rate (ranging between 10–36% of survivors). As others have reported, the chromatin environment around the target sites and sgRNAs sequence features have been identified as the major factors that affect the efficacy of CRISPR/Cas9 for any given target site^38^. As we have only targeted one gene, we have essentially assayed for only one chromatin environment that was conducive to gene editing. However, other genes may be affected negatively by different chromatin and sequence characteristics and, thus, variation in sgRNA targeting efficiency among targets differing in location across the genome is to be expected. Therefore, we recommend testing several sgRNAs for each gene to be targeted. Furthermore, in our study, mutations created in *cn* resulted in an easily scorable visible eye pigmentation phenotype which made screening of edited individuals straightforward. However, many genes of interest, such as those involved in important cellular functions, will likely yield phenotypes such as sterility, lethality, or possibly even no visible phenotype when mutated, and will, therefore, require PCR-based genotyping. Additionally, in these cases screening and selection crosses will need to be revised to obtain the mutants and maintain them (*e*.*g*., if disruption results in recessive lethal/sterile phenotypes the mutants must be maintained long term in a heterozygous state in the female sex and will require genotyping each generation).

Recently the CRISPR/Cas9 system has been demonstrated in the honey bee *Apis mellifera* ^39^, and currently other groups are developing gene editing with this system in other hymenopterans. Here we have demonstrated that CRISPR/Cas9 should be widely applicable as a feasible means for gene editing in *N*. *vitripennis*, thereby further enhancing the tractability of this haplodiploid species as an insect system for the study of important biological questions that cannot be easily addressed in other hymenopterans that are less amenable to laboratory experimentation, or in other more traditional model organisms. While not tested here, this *N*. *vitripennis* CRISPR/Cas9 approach can be later expanded to test for integration of donor constructs via homology directed repair (HDR) following CRISPR mediated cleavage, similar to other species^40–43^. This modification will allow for site specific germline transformation and will further expand the *N*. *vitripennis* tool box, given that transgenesis remains to be demonstrated, making it an even more useful model organism.

## Materials and Methods

Note - Information here provides a general overview of approaches and information on materials used, etc. A more detailed step-by-step protocol is supplied in the supplemental methods.

### Production of sgRNAs

Linear double-stranded DNA templates for all sgRNAs were generated by template-free PCR with NEB Q5 high-fidelity DNA polymerase (catalog #M0491S) by combining primer pairs (sgRNA-1F & sgRNA-R) to make sgRNA-target-1, and combining primers paris (sgRNA-2F & sgRNA-R) to make sgRNA-target-2, and combining primers paris (sgRNA-3F & sgRNA-R) to make sgRNA-target-3. PCR reactions were heated to 98 °C for 30 seconds, followed by 35 cycles of 98 °C for 10 seconds, 58 °C for 10 seconds, and 72 °C for 10 seconds, then 72 °C for 2 minutes. PCR products were purified with Beckman Coulter Ampure XP beads (catalog #A63880) according to the manufacturer protocol. Following PCR, sgRNAs were synthesized using the Ambion Megascript T7 *in vitro* transcription kit (catalog #AM1334, Life Technologies) according to the manufacturer’s protocols using 300 ng of purified DNA template overnight at 37 °C. Following *in vitro* transcription, the sgRNAs were purified with MegaClear Kit (catalog #AM1908, Life Technologies) and diluted to 1000 ng/ul in nuclease-free water and stored in aliquots at −80 °C. Recombinant Cas9 protein from *Streptococcus pyogenes* was obtained commercially (CP01, PNA Bio Inc) and diluted to 1000 ng/ul in nuclease-free water and stored in aliquots at −80 °C. Immediately prior to injection, we combined the sgRNAs (at concentrations ranging from 20–320 ng/ul) with purified Cas9 protein (at concentrations ranging from 20–320 ng/ul) in purified water and pre-blastoderm embryonic microinjections were performed. All primer sequences can be found in Table S1.

### Insect rearing, embryo collection, microinjection, transfer to hosts

*N*. *vitripennis* colonies were maintained in plastic cages (12 × 12 × 12 cm) and reared at 25 ± 1 °C with 30% humidity and a 12:12 (Light:Dark) photoperiod. Adults were fed with a 1:10 (v/v) honey/water solution that was provided in small droplets daily in a petri dish. Flesh fly pupa, *Sarcophaga bullata* (item number 144440) were ordered from www.carolina.com in batches of 100. To collect pre-blastoderm stage embryos, females and males were mated for at least 4 days. Following mating, we placed fresh *Sarcophaga bullata* pupae (hosts) into the cage to allow female wasps to parasitize the hosts for 45 minutes. Following parasitization, we carefully peeled off the puparium from the *Sarcophaga bullata* host pupae using forceps under a dissecting microscope and gently removed the recently laid exposed *N*. *vitripennis* embryos (<45 minutes old). We then quickly positioned these embryos onto a glass slide with double-sided sticky tape and injected the Cas9 protein and sgRNA mixtures into the germ cells located at the posterior of the *N*. *vitripennis* embryos. For microinjection consistency, we used a the Femtojet Express system (Eppendorf) with aluminosilicate glass filaments (Sutter Instrument). Following microinjection, we immediately placed the injected embryos back into pre-stung *Sarcophaga bullata* pupae with an ultra-fine tip paintbrush, and incubated the embryos in a humidified chamber at 25 °C until hatching.

### Cas9/gRNA-mediated mutation screens

Upon hatching, the mosaic phenotype in the G0 (injected wasps) was readily observed and assessed under microscope. Mutant individuals were isolated and mated using various crossing schemes to establish homozygous mutant stocks (Table 3). To characterize the induced mutations, genomic DNA was extracted from individual wasps with the DNeasy blood & tissue kit (QIAGEN) following the manufacturer protocol. Target loci were amplified by PCR (using primers PCR-F and PCR-R), and the PCR product was analyzed via sequencing. Mutated alleles were identified by comparison with the wild-type sequence. All photographs were obtained using fluorescent stereo microscope (Leica M165FC). Primers used for PCR and sequencing are listed in Table S1.

## Electronic supplementary material

Supplemental Protocol
