# Supplementary material for: Generation of heritable germline mutations in the jewel wasp Nasonia vitripennis using CRISPR/Cas9
Source: Sci Rep. 2017 Apr 19;7:901. doi: 10.1038/s41598-017-00990-3 (PMC5430486; doi:10.1038/s41598-017-00990-3)
Supplement: Supplementary file 1 — Supplemental Protocol [file 41598_2017_990_MOESM1_ESM.pdf]

Supplemental Protocol

Generation of heritable germline mutations in the jewel wasp *Nasonia vitripennis* using CRISPR/Cas9

Authors and Affiliations

Ming Li[1], Lauren Yun Cook Au[2], Deema Douglah[2], Abigail Chong[1], Bradley J. White[1], Patrick M. Ferree[2]* and Omar S. Akbari[1]*

[1]Department of Entomology and Riverside Center for Disease Vector Research, Institute for Integrative Genome Biology, University of California, Riverside, Riverside, CA 92521, USA

[2]W.M. Keck Science Department, Claremont McKenna, Pitzer and Scripps Colleges, 925 . Mills Avenue, Claremont, CA 91711, USA

Goals

Heritable manipulation of the *N. vitripennis* genome through precise double-strand breaks at targeted gene sites via CRISPR-Cas9. This protocol was based on several CRISPR/Cas9 protocols developed in other species including *Drosophila melanogaster*[1], *Aedes aegypti*[2], and *Danio rerio*[3].

- Efficient generation of CRISPR/Cas9 reagents: sgRNAs (Cas9 protein is commercially purchased).
- Microinjection of CRISPR/Cas9 components into *N. vitripennis* pre-blastoderm embryos.
- Isolation of mutant alleles generated by CRISPR/Cas9 mediated non-homologous end joining (NHEJ).
- Generation of stable mutant *N. vitripennis* strains through genetic crossing.

General Considerations

- It is very important to work under RNase free conditions when producing or working

with sgRNAs. Be sure to use nuclease-free consumables including filter tips and microfuge tubes. Also, thoroughly clean work area, microinjection apparatus, gloves, and pipettes with RNaseZap (Ambion) before conducting experiments.

- Once sgRNAs are produced, immediately mix these reagents with Cas9 protein at the final desired injection concentrations and make small 5-10ul aliquots. Store these ready-to-inject final mixtures at -80°C until needed. The goal here is to avoid excess freeze-thaw-cycles for both the sgRNAs and the Cas9 protein as much as possible.
- Given that not all sgRNAs function efficiently, and specificity and activity are unpredictable, we recommend designing multiple sgRNAs for each target gene to increase probability of generating desired modifications of target genes.
- To collect enough *N. vitripennis* eggs for microinjection, it is important to expand stock wasp colonies to sufficient numbers. We recommend to establish 2-3 colonies with 200-500 wasps (of both sexes mixed) per colony.

Selected Kits and Reagents

- *Streptococcus pyogenes* Cas9 recombinant protein, PNA Bio (catalog # CP01)
- Flesh fly pupa, *Sarcophaga bullata,* www.carolina.com (catalog# 144440)
- Beckman Coulter Agencourt Ampure XP beads (catalog #A63880)
- Zymo Research DNA Clean and Concentrator kit (catalog #D4005)
- NEB Q5 High-Fidelity DNA polymerase (catalog # M0491S) - PCR of sgRNA
- Ambion MegaScript T7 (catalog #AM1334) – sgRNA *in vitro* transcription kit
- Ambion MegaClear Kit (catalog #AM1908) – Purification of *in vitro* transcribed sgRNA
- Agilent Bioanalyzer 2100 and RNA 6000 Nano Kit (catalog #5067-1511)
- Ambion RNaseZap (catalog # AM9780) - removal of RNase's from work area.
- DNeasy blood & tissue kit (QIAGEN, catalog # 69506)- genomic DNA isolation
- dNTP solution mix, 25 mM each (Enzymatics, catalog # N205)
- UltraPure DNase/RNase-free distilled water (Life Technologies, catalog # 10977-023)
- Double-sided sticky tape
- 10% (vol/vol) sucrose solution

- Microloader Tips for Filling Femtotips (Eppendorf, catalog # 930001007)
- sgRNA-F and sgRNA-R primers from IDT as PAGE-purified oligos

Equipment

We recommend the following equipment, although each item can be substituted by others, depending on a given lab's set-up.

- Sutter instruments Microelectrode Beveler (Model # BV10)
- World Precision Instruments (WPI) micromanipulator (Model # Kite R)
- Eppendorf Femtojet Express programmable microinjector
- Sutter micropipette puller (P-1000, and P-2000)
- Olympus SZ51 -screening microscope
- Leica DM-750 -microinjection microscope
- Several bugdorm-41515 (L17.5 x W17.5 x H17.5 cm) cages
- Microinjection needles
    - Borosilicate glass capillary tubing 1 mm (outside diameter) × 0.58 mm (inner diameter) (Sutter Instrument, catalog # BF100-58-10)
    - Quartz glass capillary tubing 1 mm (outside diameter) × 0.70 mm (inner diameter) (Sutter Instrument, catalog # QF100-70-10)
    - Aluminosilicate glass capillary tubing 1 mm (outside diameter) × 0.64 mm (inner diameter) (Sutter Instrument, catalog # AF100-64-10)
- PCR Machine with heated lid (BIO-RAD, T100^{TM})

Genomic DNA target site design and selection criteria

The revolutionary CRISPR/Cas9 gene editing system relies on the target sequence encoded by an engineered sgRNA to guide the Cas9 nuclease to a desired genomic target location, allowing for base-pairing interactions between the sgRNA/Cas9 complex and the complementary genomic DNA sequence, thereby resulting in subsequent Cas9 mediated cleavage of the specified genomic target sequence. The characterized recognition sequence for the *Streptococcus pyogenes* Cas9 protein relies on the presence of a protospacer associated motif, or PAM, to be located

immediately adjacent to the desired genomic target sequence[4–7]. The PAM sequence is NGG, which is located in the genome directly downstream of the desired 20bp genomic target sequence, taking the form N20-NGG. Importantly, the PAM sequence is not included in the either the sgRNA template DNA, or the *in vitro* transcribed sgRNA. To define putative sgRNA genomic target sites we suggest to consider several factors. First, confirming transcriptional expression of the target region, and looking for conservation between other species, will help define putative *N. vitripennis* genomic target regions that have a higher probability of being necessary for gene function (assuming the goal is to disrupt gene function). To do this we recommend using both available *N. vitripennis* transcriptional databases and simple NCBI-BLAST searches (www.vector.caltech.edu)[8,9]. Secondly, once general target regions are defined, the putative sgRNA target sites can be identified by simply scanning both the sense and antisense strands for the presence of the NGG-PAMs either manually by eye, or by utilizing available software such as CHOPCHOP v2[10] , and/or local sgRNA Cas9 packages[11]. Finally, to minimize potential off-target effects, we recommend confirming specificity of the sgRNAs using publicly available bioinformatic tools for example NCBI-Blast[12], Blat[13] and selecting the most specific sgRNAs within the specified target regions with the least potential off-target binding sites. It should be noted that even if the chosen sgRNA target sequences fulfill all of the above requirements, sgRNA specificity and activity is unpredictable. Therefore, we recommend that multiple different sgRNAs are designed to target the exonic coding sequences and co-injected in order to increase the chance of editing a target gene of interest.

Shown below is an example of a genomic target region depicted in figure 1 with three chosen sgRNA target sequences. The primary genomic target sequences are highlighted in **green**, while the PAMs are highlighted in **blue** (**NGG**).

- Target # 1: **ACATTACATCGGAATCGTACCGG**
- Target # 2: **GGATCCTCATGACATTACATCGG**
- Target # 3: **GATTCCGATGTAATGTCATGAGG**

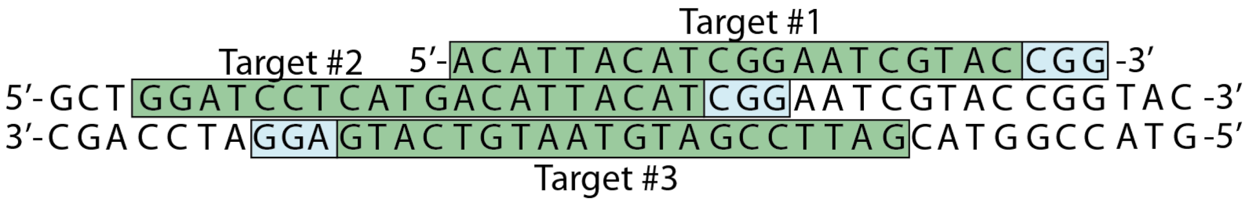

Figure 1 . A genomic DNA example target region with target sites (#1-3) highlighted in green and the PAM sequences are highlighted in blue. Targets sites #1&2 are located on the top strand, while target site # 3 is located on the bottom strand.

Importantly, the first two nucleotides transcribed by the T7 RNA polymerase should ideally be "GG," and therefore, it is important that the *in vitro* transcribed sgRNA sequences beings with these two nucleotides. Therefore, if perhaps the chosen sgRNA sequences (**green**) do not begin with "GG," these nucleotides can be added onto the 5' end of the target sequence. For example, for the target sequences highlighted in figure 1, the following bases (**pink**) would be added to ensure robust *in vitro* transcription by the T7 RNA polymerase. For target # 1, two GG's would need to be added, for target # 2 no extra GG's would need to be added since it already begins with a GG, and for target # 3 one G would need to be added to ensure the first two nucleotides (underlined) begin with "GG."

- Target # 1: **GG ACATTACATCGGAATCGTACCGG**
- Target # 2: **GGATCCTCATGACATTACATCGG**
- Target # 3: **GGATTCCGATGTAATGTCATGAGG**

sgRNA DNA template generation

The first step in sgRNA synthesis is to produce the template DNA to be used for *in vitro* transcription. This DNA is generated by template-free PCR using two primers that anneal to each other via complementary sequences (**bold and underlined**). These primers can be ordered from IDT as PAGE-purified oligos.

sgRNA-R (Table S1) - This is a universal reverse primer that can be used to generate all sgRNA targets containing the sgRNA backbone sequence.

- 5’-AAAAGCACCGACTCGGTGCCACTTTTTCAAGTTGATAACGGACTAGCCTTATTTTAACTT**GCTATTTCTAGCTCTAAAAC**-3’

sgRNA-F - This primer contains the T7 promoter upstream (**orange**) of the target sequence.

- 5’–**GAAATTAATACGACTCACTATA GG**$\mathbf{N^{20}}$ **GTTTTAGAGCTAGAAATAGC** –3’

Note: The **GG**$\mathbf{N^{20}}$ is a generic sequence that includes the 20 nucleotide user-defined genomic target sequence ($\mathbf{N^{20}}$), and the “GG” sequence necessary for i*n vitro* T7 RNA polymerase transcription, but does **NOT** include the PAM sequence.

1. The first step in sgRNA production is to setup a template-free PCR using sgRNA-R and sgRNA-F primers to produce the linear dsDNA templates that will be used for *in vitro* transcription reactions. We prefer to use NEB’s Q5 high-fidelity DNA polymerase, however other DNA polymerases should also work. Below is the PCR reaction we recommend.

<table>
<tr><th colspan="2">PCR Reagents</th><th>PCR Program</th></tr>
<tr><td>5x Q5 Reaction Buffer</td><td>20ul</td><td rowspan="7">Initial Denaturation Step
98℃ - 30 Seconds
Amplification
35 cycles:
98℃- 10 seconds (Denaturation)
58℃- 10 seconds (Primer Anneal)
72℃- 10 seconds (Extension)
Final Extension
72℃- 2 minutes
Storage
4℃- ∞</td></tr>
<tr><td>10 mM dNTPs</td><td>2ul</td></tr>
<tr><td>10 uM Forward Primer (sgRNA-F)</td><td>5ul</td></tr>
<tr><td>10 uM Reverse Primer (sgRNA-R)</td><td>5ul</td></tr>
<tr><td>Q5 High-Fidelity DNA Polymerase</td><td>1ul</td></tr>
<tr><td>Nuclease-Free Water</td><td>67ul</td></tr>
<tr><td>Total</td><td>100ul</td></tr>
</table>

2. Following the PCR reaction, run 3ul of the PCR product on a DNA agarose gel (2%) to confirm the PCR reaction amplified the desired DNA and the size (band ~122bp in size) is as expected.
3. The PCR products are then purified with Beckman Coulter Ampure XP beads (catalog #A63880) according to the manufacturer protocol. Note - instead of the Ampure XP beads, we have also used the Zymo DNA clean and Concentrator kit at this step and that also effective.

   Simplified Beckman Coulter Ampure XP beads protocol:

   a. Gently shake the Beckman Coulter Ampure XP bottle to resuspend the magnetic particles.
   b. Add 1.8x volume of Ampure XP beads to the PCR reaction in a 1.5 ml microcentrifuge tube.
   c. Mix reagents and PCR reaction thoroughly by pipetting up and down.
   d. Incubate for 5 min at room temperature for maximum recovery.
   e. Place the tube on a magnetic rack and wait until the liquid is clear to capture the beads. Carefully remove supernatant.
   f. Keep the tube on the magnetic rack and add 300 ul freshly made 80% ethanol (make with nuclease-free water) to wash the beads.
   g. Incubate the tube at room temperature and wait for 1 min.
   h. Carefully remove supernatant.
   i. Repeat steps f-h again.
   j. Air dry the beads at room temperature for 5 min.
   k. Remove the tube form the magnetic rack.
   l. Resuspend the beads in 20 ul of nuclease-free water.
   m. Incubate the tube at room temperature for 10 min to elute DNA from the beads.
   n. Place the tube back to the magnetic rack until the liquid is clear.
   o. Transfer supernatant to a new tube.

Simplified Zymo DNA clean and Concentrator kit protocol:

- **a.** In a 1.5 ml tube, add 5 volumes of DNA Binding Buffer to each volume of PCR reaction.
- **b.** Mix reagent and PCR reaction thoroughly by pipetting.
- **c.** Transfer mixture to a Zymo-Spin^{TM} Column in a collection tube.
- **d.** Centrifuge for 30 seconds (11000 x g) and discard the flow-through.
- **e.** Add 200 ul DNA wash buffer to the column, and centrifuge for 30 seconds (11000 x g), discard the flow-through.
- **f.** Repeat the step e again.
- **g.** Add 20 ul nuclease-free water directly to the column matrix.
- **h.** Incubate at room temperature for 1 min.
- **i.** Transfer the column to a new 1.5 tube and centrifuge for 1 min (11000 x g) to elute the DNA.

4. Following purification, measure the purity and concentration of the purified DNA template using a nanodrop. We aim to have a concentration of over 100 ng/ul to ensure enough template for the *in vitro* transcription reaction.

sgRNA production by *in vitro* transcription

To produce the sgRNAs, we use the Ambion MegaScript T7 *in vitro* transcription kit and followed the manufacturer's protocol.

1. Briefly, we thaw and mix thoroughly the ribonucleotides (keep on ice) and reaction buffer (keep at room temperature), then add all reagents to a PCR tube in the following order.

***In vitro* Transcription Reagents**	**Volume**	**Reaction**
Nuclease free water	To 20uL	2 hours (minimum) to overnight (12-16
Free ribonucleotides (ATP, CTP, GTP, UTP)	2ul each (8ul total)	

10x Reaction Buffer	2ul	hours maximum) @ 37℃. Note - we recommend overnight incubation here.<br><br>Add 1ul of Turbo DNase (mix well) and incubate @ 37℃ for 15 minutes.
sgRNA PCR template	300ng total (~3ul)	
T7 Enzyme	2ul	
**Total**	**20ul**	

5. Following *in vitro* transcription, 1ul of Turbo DNase should be added to the reaction and incubated @ 37℃ for 15 minutes to remove the template DNA from the reaction. The sgRNAs can then be purified with Ambion MegaClear Kit following the manufacturer protocol.

Simplified Ambion MegaClear protocol

a. In a 1.5 ml tube, bring the RNA sample to 100 ul with the elution solution. Mix gently but thoroughly by pipetting.
b. Add 350 ul of binding solution concentrate to the sample. Mix gently but thoroughly by pipetting.
c. Add 250 ul of 100% ethanol to the sample. Mix gently but thoroughly by pipetting.
d. Pipet the RNA mixture above onto the filter cartridge and centrifuge for 1 min at RCF 13000 x g.
e. Discard the flow-through.
f. Wash with 500 ul wash solution, discard the flow-through.
g. Repeat the step f.
h. After discarding the wash solution, centrifuge the filter cartridge for 1 min at RCF 13000 x g.
i. Place the filter cartridge into a new 1.5 ml tube.
j. Add 50 ul of nuclease-free water to the center of the filter cartridge.

k. Close the cap of the tube and incubate at 70°C for 10 min.

l. Centrifuge (13000 x g) for 1 min at room temperature to elute RNA.

6. The final concentration should be measured using a nanodrop, and quality can be measured with an Agilent Bioanalyzer confirming that sgRNA appears as a single band without any degradation products.
7. sgRNAs can then be diluted to 1000 ng/ul in nuclease-free water and stored in aliquots @ -80°C. We generally produce roughly 5-100ug of sgRNA from this reaction depending on the template DNA quality.

Preparation of sgRNA/Cas9 mixtures for microinjection

Before microinjection the purified recombinant Cas9 protein from *Streptococcus pyogenes* should be obtained commercially (CP01, PNA Bio Inc) and diluted to 1000 ng/ul using UltraPure DNase/RNase-free distilled nuclease free water and stored @ -80°C.

- This stock Cas9 protein solution should be diluted with nuclease free water and mixed with the purified sgRNAs at various concentrations (20-320 ng/ul) in small 5-10ul aliquots.
- These ready-to-inject final mixtures can be stored at -80C until needed. The goal here is to avoid excess freeze-thaw-cycles for both the sgRNAs and the Cas9 protein as much as possible.
- For *N. vitripennis*, we found the optimal concentrations for both the Cas9 protein and purified sgRNAs to be 160 ng/ul for each component.
- To prepare these mixtures thaw and mix both components in UltraPure DNase/RNase-free distilled nuclease free water on ice, and maintain these mixtures on ice while performing injections.

Preparation of needles for *N. vitripennis* embryo microinjection

For effective penetration and microinjection into *N. vitripennis* eggs, we experimented with several types of capillary glass needles with filament including Quartz, Aluminosilicate and Borosilicate types. The quality of needles is critical for avoiding breakage/clogging during injection, embryo survival and transformation efficiency. For each of these glass types we

developed effective protocols to pull these needles on different Sutter micropipette pullers (P-1000, and P-2000) to enable the needles to have a desired hypodermic-like long tip that we found effective for *N. vitripennis* embryo microinjection. The parameters (filament, velocity, delay, pull, pressure) for the different types of capillary glass needles are listed in the following table. While all three types of needles were effective for *N. vitripennis* injections, we prefered the Aluminosilicate capillary glass needles, because the Quartz capillary glass needles were too expensive, and the Borosilicate capillary glass needles were a bit too soft and clogged easily.

Capillary glass needle type	Sutter Needle Puller model	Heat	Filament	Velocity	Delay	Pull	Pressure
**Quartz**	P-2000	750	4	40	150	165	-
**Aluminosilicate**	P-1000	605	-	130	80	70	500
**Borosilicate**	P-1000	450	-	130	80	70	500

***N. vitripennis* pre-blastoderm stage embryos collection and alignment**

1. Before collecting embryos, it is important to expand *N. vitripennis* colonies and set up several (3-4) bugdorm-41515 (L17.5 x W17.5 x H17.5 cm) cages with roughly 200-500 adult wasps in each cage (figure 2). This will ensure enough eggs are laid on demand for microinjection. Note: population cages are not necessary; wasps can be maintained in smaller numbers in multiple glass tubes (10 mL or larger in volume) and plugged with

cotton.

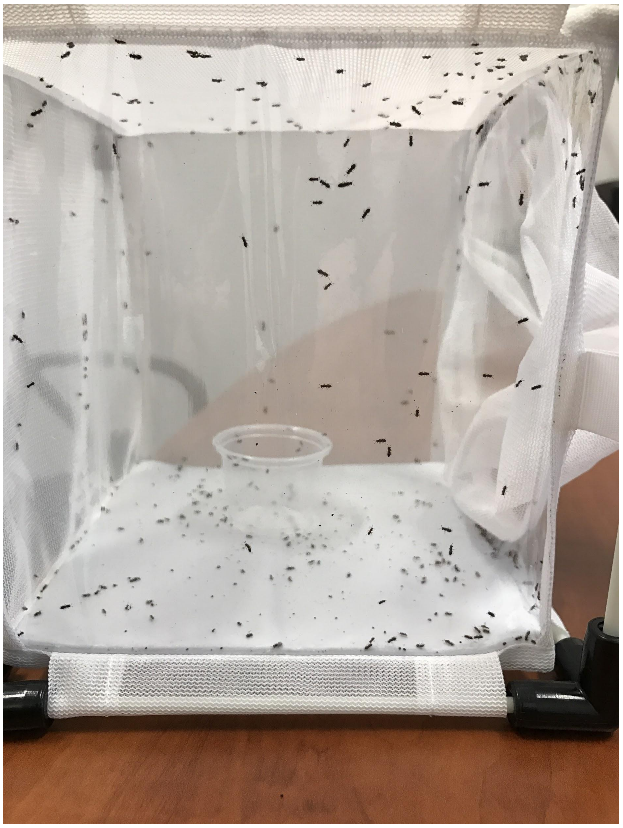

Figure 2. *N. vitripennis* colony in a bugdorm-41515 (L17.5 x W17.5 x H17.5 cm) with roughly 200-300 adult wasps.

2. Make sure the wasps are healthy, and well fed, by freshly providing small droplets of 50:50 (v/v) honey/water solution daily, and removing old honey/water solution. Maintain *N. vitripennis* colonies at 25 ± 1 °C with 30% relative humidity and a 12:12 (Light : Dark) photoperiod.
3. Allow the females and males to freely mate for at least 4 days, prior to injection, and keep them completely starved of hosts to ensure females lay eggs when needed.
4. When ready to collect embryos, place a few (2-3) fresh *Sarcophaga bullata* pupae into the cage or vial with the gravid wasps. Importantly, use a foam stopper (with a pupal-sized hole in a foam stopper made using a ballpoint pen) to only expose only about 0.5 cm of the hosts for parasitization to ensure that the embryos are laid in a concentrated manner at the posterior end of the host for rapid egg collection (figure 3). Alternatively,

a 1-ml pipette tip cut ~0.5 cm from the end can also be used to restrict egg laying on host as described previously[14].

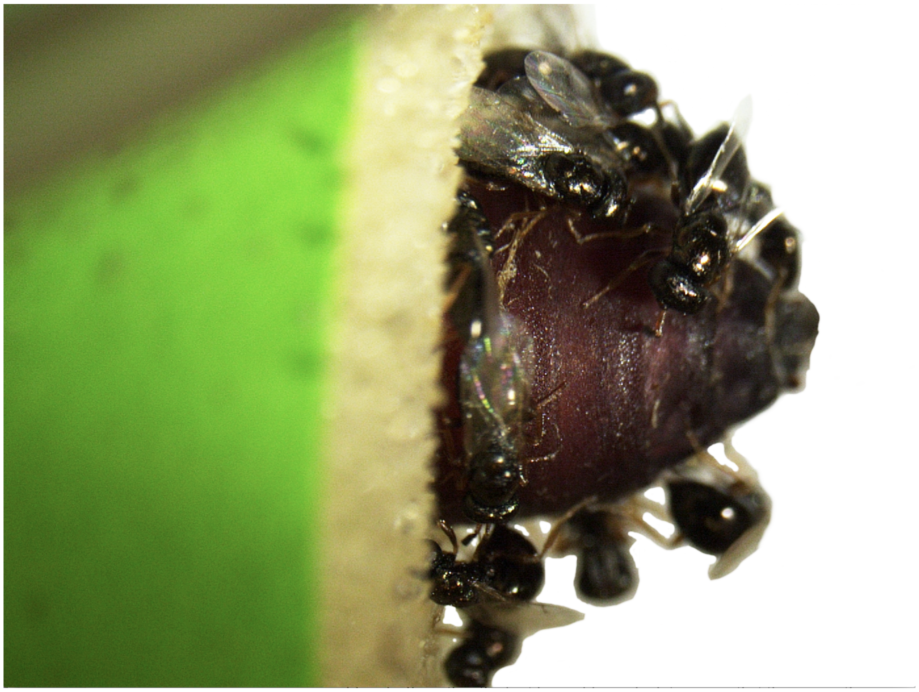

Figure 3. Gravid *N. vitripennis* females parasitizing 0.5cm of the posterior end of a *Sarcophaga bullata* pupae. Importantly, each pupa is placed inside of a foam stopper (green) to ensure embryos are laid in a concentrated manner at the posterior end.

5. Allow female wasps to parasitize (oviposit embryos) the host for roughly 30 minutes at 25℃. Then remove the host and replace with a new host, every 15 minutes, to ensure sufficient eggs for continuous injection. Note - it is very important that the embryos are as young as possible, ideally within the first hour of being oviposited, to ensure that they are in the pre-blastoderm stage. Old embryos (>1.0 hour) should not be injected.
6. To collect embryos, remove parasitized hosts from the foam stopper. Under a dissecting microscope, carefully peel off the posterior end of the puparium that was exposed to the wasps using forceps. Embryos will be resting on the surface of the host pupa (figure 4). Carefully remove embryos from host, using a fine-tip wet paintbrush, ensuring not to burst the soft pupal skin inside the host. Note: Ultrafine tweezers can also be used to gently pick off the embryos; however, care must be taken in this particular case to not

burst the embryos or the host pupa.

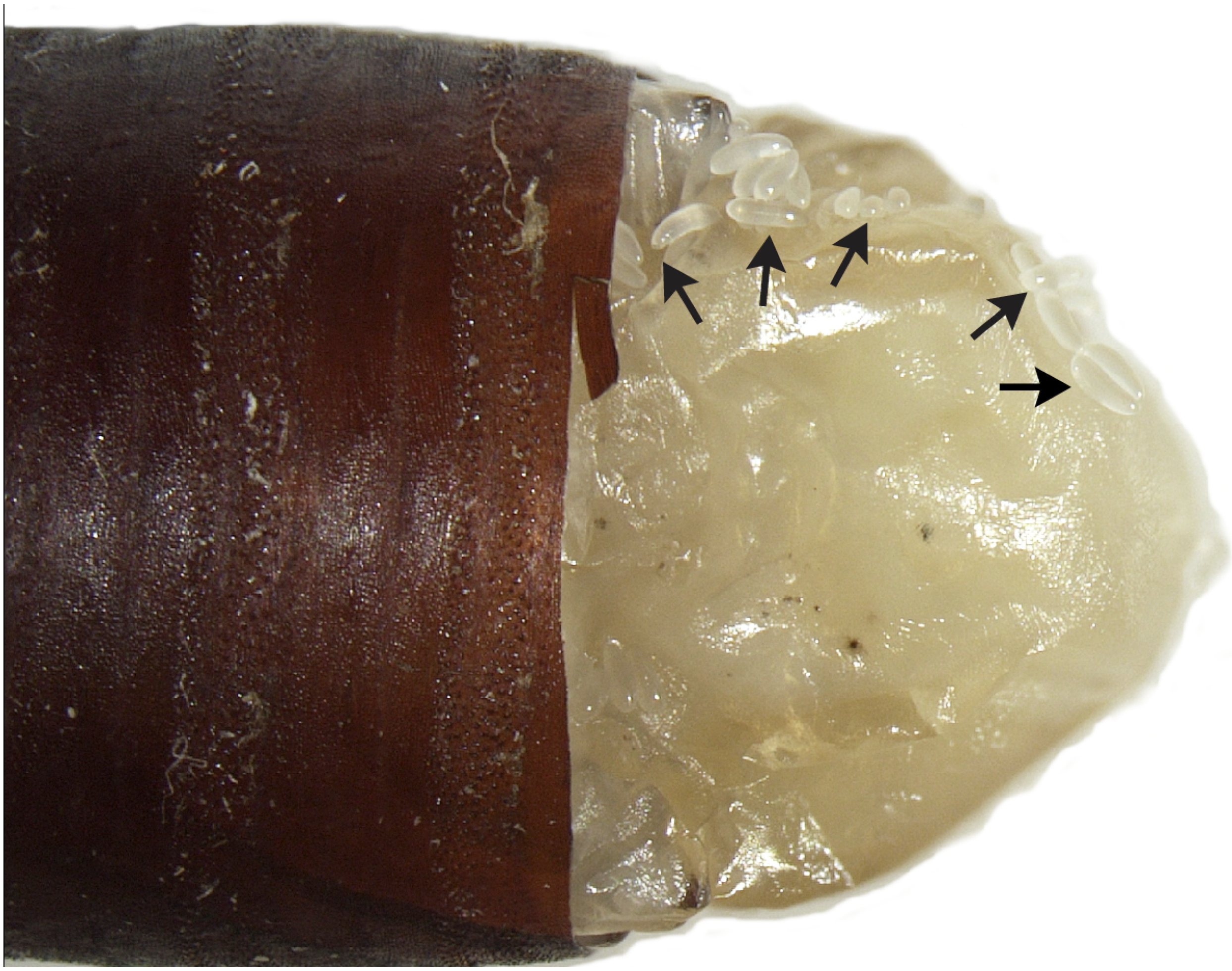

Figure 4. Parasitized *Sarcophaga bullata* pupae by *N. vitripennis*. The puparium has been peeled off at the posterior end using forceps, thereby exposing the *N. vitripennis* eggs (black arrows pointing to embryos).

7. Transfer embryos one-by-one to double-sided sticky tape (fixed to a glass slide). Using a wet paintbrush (or single side of a pair of ultrafine forceps) orient the eggs one-by-one in a row so the posterior end (more narrow end) is pointing in the same direction for each egg (figure 5). Note - we found embryo survival rates to be greater if we did not cover eggs with halocarbon oil during injection as is done for *Drosophila melanogaster* microinjection[15]. Since oil is not used, it is important to keep the embryos moist during the injection period by regularly adding water using the paintbrush. The amount of water

on the brush is key to move embryos around and align with ease. Too much water results in embryos floating and not sticking well to the double-sided sticky tape, and too little water makes them difficult to move around and can lead to desiccation. To adjust the degree of moisture, dip the tip of the brush in water then lightly touch the tip of the brush to a dry kimwipe.

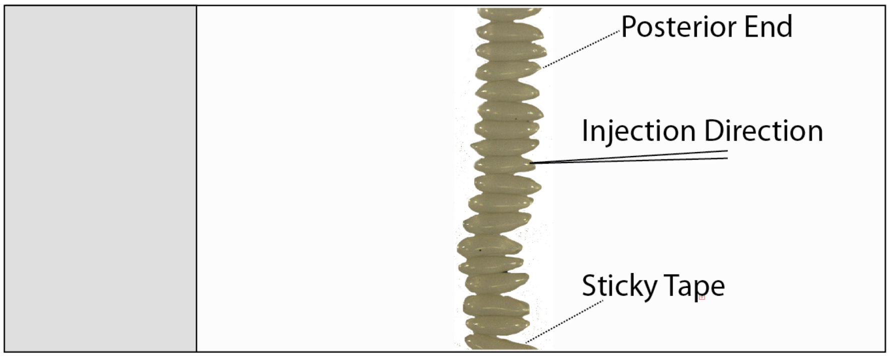

Figure 5. *N. vitripennis* embryos are lined up on a glass slide on a piece of sticky tape, and injected one-by-one into the posterior end.

8. Ideally this protocol is most effective if one person is continuously collecting and lining up eggs, while another person is injecting the CRISPR/Cas9 components.

CRISPR/Cas9 embryo Microinjection

1. Break the closed tip of the needle by either rubbing the needle to the edge of the slide, or by using a microelectrode beveler (Sutter Instrument).
2. Load the needle with 2ul of injection mixture using Microloader Tips for Filling Femtotips.
3. One-by-one inject, 1-5pl of injection mixture (about 2 -10% of the egg's volume) into the cytoplasm from the posterior end of each egg. We use a femtojet express to control for the injection volume and an old-fashioned manual micromanipulator for embryo injection (World Precision Instruments Kite-R).
4. Inject ~40 eggs at a time (should take roughly 10 minutes) then stop and transfer injected eggs into a host then continue injecting again using a fresh newly laid batch of eggs. Note: Depending on needle, needle clogging will likely be an issue. In our experience,

the needle would clog once in every 25 embryo injections, and we would either re-bevel the needle or would need to use a new needle.

Transferring embryos back to the hosts

1. Following microinjection, transfer injected embryos back into a pre-stung *Sarcophaga bullata* pupae using a fine-tip paintbrush (figure 6) or ultrafine forceps. *N. vitripennis* larva utilize the host pupa as a food source to complete larval development and to our knowledge there is currently no available artificial diet that can be used.
2. Very important - be sure to **only** transfer eggs back into a **pre-stung host**, otherwise embryos will not survive. When a female wasp stings a host, she uses her ovipositor to bore a hole in the host puparium to inject venom which causes arrest of the pupal development and begin tissue necrosis, allowing the *N. vitripennis* larvae to consume the host. Without the venom, the host will survive and the *N. vitripennis* larvae will not be able to consume the host.
3. To ensure a host has already been stung, find a host with embryos in it, then scrape all the embryos off and use it as the host. Also, to avoid overcrowding, only place about 40 injected embryos or less per host.

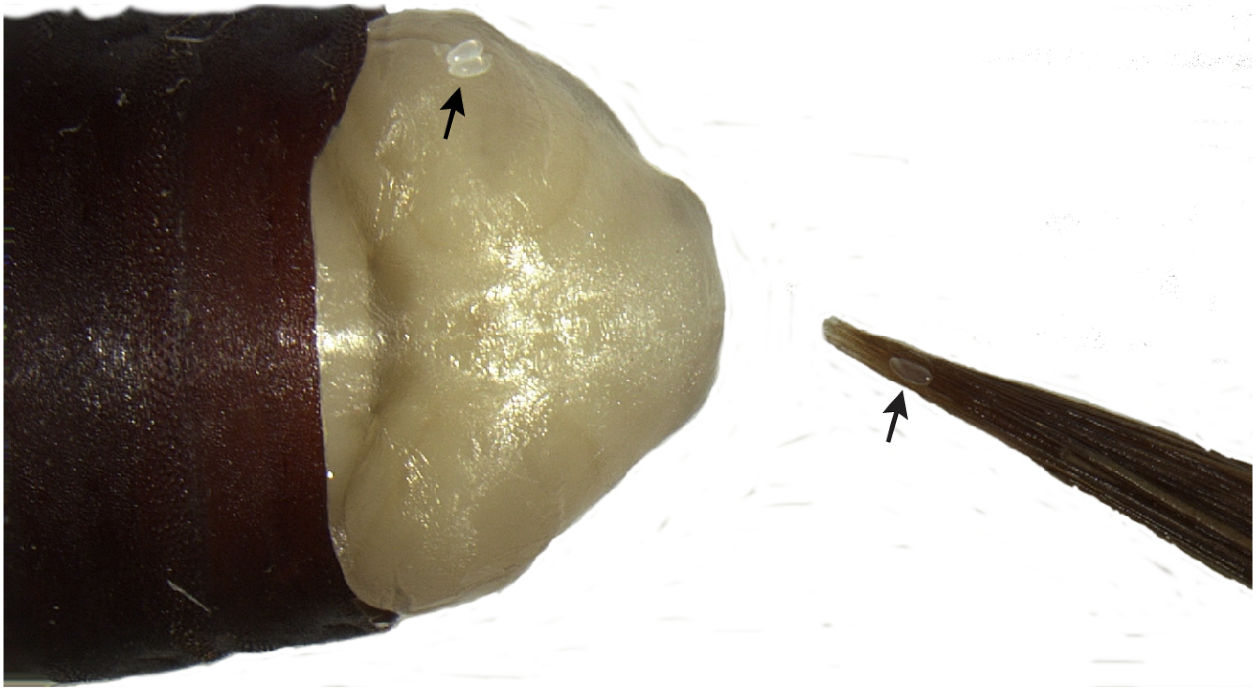

Figure 6. Injected *N. vitripennis* embryos are individually transferred to a pre-stung *Sarcophaga bullata* pupae using a wet fine-tip paintbrush. The puparium has been peeled off at the posterior end using forceps, to allow placement of the *N. vitripennis* embryos (black arrows pointing to embryos).

4. Incubate hosts harboring transferred injected eggs in a moist humidified chamber (e.g. petri dish with cotton balls moist with water) at 25°C until hatching (roughly 1-2 days). Importantly, hosts can be left with a peeled off puparium and the *N. vitripennis* eggs will develop normally so long as they are incubated in a humidified chamber (petri dish with damp filter paper and cotton balls) with roughly 70% relative humidity (figure 7) .

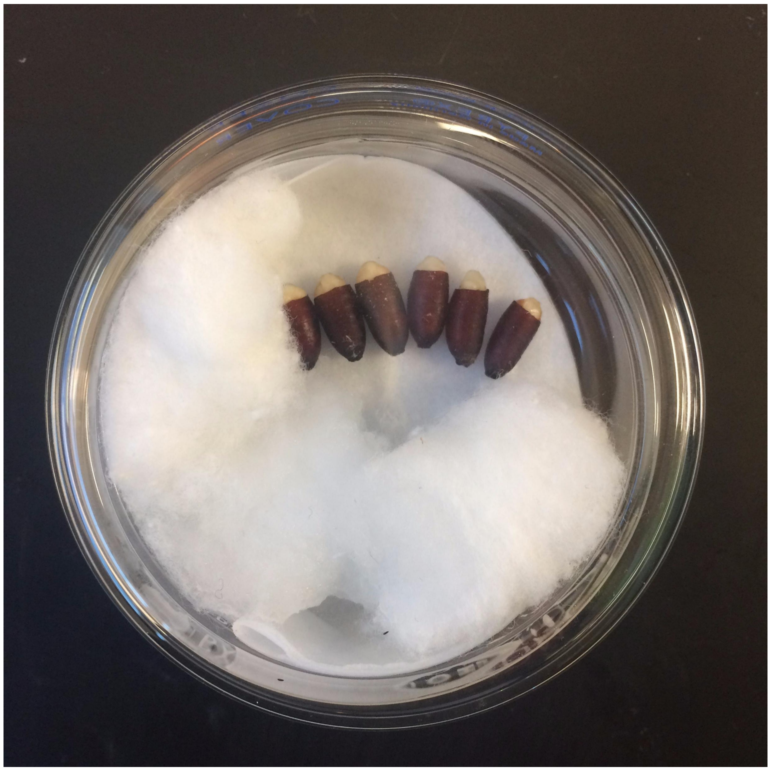

Figure 7. Incubation of *Sarcophaga bullata* hosts harboring the injected *N vitripennis* eggs in petri dish for roughly 2 weeks.

5. Monitor the embryos, the hatched *N vitripennis* larvae, and the host daily. Remove any dead *N. vitripennis* larvae, and if the host becomes infected with bacteria or dies (turns to the gray or dark color and has a foul smell) transfer the larvae to a fresh pre-stung host.

<u>Genetic crosses and screening for mutations</u>

1. After roughly 8 days the injected embryos will begin to pupate. Once they pupate they will no longer consume food (i.e. blowfly host) and can be removed from the host.
2. Remove each *N. vitripennis* pupae from the host, and place in an individual 1.5ml eppendorf tube until hatching. This will ensure that the hatched females will be virgin and will not mate until desired.
3. Upon hatching, if disrupting a visual marker gene (e.g. *cinnabar*) then the mutant phenotype should be readily visible, given that full eye pigmentation is complete by late pupal stages. If disrupting a non-visible marker gene, then every surviving G0 (injected

individual) should be mated to a couple of wild type wasps of the opposite sex individually (to virgin females in the case of mating G0 injected males), and given a separate host to produce its own colony. Importantly, similar to *Drosophila melanogaster*, *N. vitripennis* can be immobilized by exposure to CO_2 allowing for straightforward manipulation.

4. Once the injected G0 males and females and have successfully mated, and produced progeny, the G0's can be sacrificed and genomic DNA should be extracted using the DNeasy blood & tissue kit for every individual.

Simplified DNeasy blood & tissue kit Protocol

- **a.** Place the sample into a sterile 1.5 ml microcentrifuge tube.
- **b.** Add 180 ul buffer ATL and 20 ul proteinase K, mix by vortexing 10-15 seconds.
- **c.** Incubate the sample overnight at 56 °C until completely lysed.
- **d.** Add 200ul buffer AL. Mix thoroughly by vortexing.
- **e.** Add 200ul ethanol (96%-100%). Mix thoroughly by vortexing.
- **f.** Pipet the mixture into a DNeasy mini spin column placed in a 2 ml collection tube.
- **g.** Centrifuge at 8000 x g for 1 min.
- **h.** Discard the flow-through and collection tube. Place the spin column in a new 2 ml collection tube.
- **i.** Add 500 ul buffer AW1. Centrifuge for 1 min at 8000 x g.
- **j.** Discard the flow-through and collection tube. Place the spin column in a new 2 ml collection tube.
- **k.** Add 500 ul buffer AW2, and centrifuge for 3 min at 20000 x g.
- **l.** Discard the flow-through and collection tube. Transfer the spin column to a new 1.5 ml tube.
- **m.** Add 30 ul buffer AE to the center of the spin column membrane.
- **n.** Incubate for 1 min at room temperature.
- **o.** Centrifuge for 1 min at 8000 x g.

5. The presence of mutations can be determined by PCR amplifying/sequencing the

genomic target region.

6. Colonies that have mutations as determined by sequencing should be continued, while colonies that were established with non-mutant G0's should be discarded.
7. Importantly, unmated females will give rise to 100% haploid male broods, so therefore a mutant unmated female can give rise to large number of mutant males that can be used for subsequent analysis. Note: mutations in essential genes will need to be kept by mating surviving G0 injected females (presumably heterozygous for a mutation) to wild type males; in this particular case, half of the F1 male progeny should die due to inheritance of the lethal mutation, while half of the F1 female progeny will be carriers of the lethal mutation.

Table S1. Primers used in this study.

Primer	Primer sequence (5'-3')*
**PCR-F**	GACTGGTGCGTAACATTTCATC
**PCR-R**	AGCGAGACTCGAGCAATAAC
**sgRNA-1F**	GAAATTAATACGACTCACTATAGGGTAGGGAAGCTCTGAGGGGTTTTAGAGCTAGAAATAGC
**sgRNA-2F**	GAAATTAATACGACTCACTATAGGCGGTGGGCTTGGAAGACGGTTTTAGAGCTAGAAATAGC
**sgRNA-3F**	GAAATTAATACGACTCACTATAGGAGCTTGTTCAGATGGGTTGTTTTAGAGCTAGAyAATAGC
**sgRNA-R**	AAAAGCACCGACTCGGTGCCACTTTTTCAAGTTGATAACGGACTAGCCTTATTTTAACTTGCTATTTCTAGCTCTAAAAC

*text underlined in blue represents the sgRNA target site in *cn*.

Supplemental References

1. Bassett, A. R., Tibbit, C., Ponting, C. P. & Liu, J.-L. Highly efficient targeted mutagenesis

of Drosophila with the CRISPR/Cas9 system. *Cell Rep.* **4,** 220–228 (2013).

2. Kistler, K. E., Vosshall, L. B. & Matthews, B. J. Genome engineering with CRISPR-Cas9 in the mosquito Aedes aegypti. *Cell Rep.* **11,** 51–60 (2015).
3. Hwang, W. Y. *et al.* Efficient genome editing in zebrafish using a CRISPR-Cas system. *Nat. Biotechnol.* **31,** 227–229 (2013).
4. Mali, P., Esvelt, K. M. & Church, G. M. Cas9 as a versatile tool for engineering biology. *Nat. Methods* **10,** 957–963 (2013).
5. Ran, F. A. *et al.* Genome engineering using the CRISPR-Cas9 system. *Nat. Protoc.* **8,** 2281–2308 (2013).
6. Wiedenheft, B., Sternberg, S. H. & Doudna, J. A. RNA-guided genetic silencing systems in bacteria and archaea. *Nature* **482,** 331–338 (2012).
7. Hsu, P. D. *et al.* DNA targeting specificity of RNA-guided Cas9 nucleases. *Nat. Biotechnol.* **31,** 827–832 (2013).
8. Akbari, O. S., Antoshechkin, I., Hay, B. A. & Ferree, P. M. Transcriptome profiling of Nasonia vitripennis testis reveals novel transcripts expressed from the selfish B chromosome, paternal sex ratio. *G3* **3,** 1597–1605 (2013).
9. Ferree, P. M. *et al.* Identification of Genes Uniquely Expressed in the Germ-Line Tissues of the Jewel Wasp Nasonia vitripennis. *G3* **5,** 2647–2653 (2015).
10. Labun, K., Montague, T. G., Gagnon, J. A., Thyme, S. B. & Valen, E. CHOPCHOP v2: a web tool for the next generation of CRISPR genome engineering. *Nucleic Acids Res.* **44,** W272–6 (2016).
11. Xie, S., Shen, B., Zhang, C., Huang, X. & Zhang, Y. sgRNAcas9: a software package for designing CRISPR sgRNA and evaluating potential off-target cleavage sites. *PLoS One* **9,**

e100448 (2014).

12. Stover, N. A. & Cavalcanti, A. R. O. in *Current Protocols Essential Laboratory Techniques* 11.1.1–11.1.35 (2014).
13. Bhagwat, M., Young, L. & Robison, R. R. Using BLAT to find sequence similarity in closely related genomes. *Curr. Protoc. Bioinformatics* **Chapter 10,** Unit10.8 (2012).
14. Lynch, J. A. & Desplan, C. A method for parental RNA interference in the wasp Nasonia vitripennis. *Nat. Protoc.* **1,** 486–494 (2006).
15. Spradling, A. C. & Rubin, G. M. Transposition of cloned P elements into Drosophila germ line chromosomes. *Science* **218,** 341–347 (1982).
